# A Bibliometrics Analysis of Metformin Development From 1980 to 2019

**DOI:** 10.3389/fphar.2021.645810

**Published:** 2021-04-28

**Authors:** Yanjun Song, Pei Ma, Yu Gao, Peigen Xiao, Lijia Xu, Haibo Liu

**Affiliations:** Institute of Medicinal Plant Development, Chinese Academy of Medical Sciences, Peking Union Medical College, Beijing, China

**Keywords:** metformin, bibliometrics analysis, development process, research frontier, Web of Science

## Abstract

Metformin, the first-line oral blood glucose-lowering agent to manage type 2 diabetes, has gained growing popularity on both clinical application and basic research since early 1980s. A thorough and systematic knowledge map of metformin is pertinent to evaluate the research frontier and determine knowledge gaps. To this end, 20, 526 publications were analyzed by bibliometrics and data visualization to demonstrate the current global research status, potential hotspots, and perspectives on future research directions. In addition, the metformin development along the historical line was illustrated over the last 40 years. In sum, this study provides a comprehensive analysis that delineates the evolution of the historical milestones of metformin development, and we discuss the future research directions based on objective data analysis from a wide spectrum of metformin research areas.

## Introduction

Metformin (dimethylbiguanide), a guanidine derivative first extracted from *Galega officinalis*, is the first-line drug for treatment of type 2 diabetes mellitus, due to its high efficacy and safety in glycemic control (Graham et al., 2011), with clear, however, incompletely understood cardioprotective benefits (Zilov et al., 2019). Biguanides regulate hepatic gluconeogenesis mainly *via* decreases in cellular energy charge and subsequent activation of AMPK signaling. Recent emphasis is given to the possible alteration of the intestinal microbiota by metformin (Buse et al., 2009). The latest studies suggest the repurposing potential of metformin for cancer (Aljofan and Riethmacher, 2019), age-related diseases (Kulkarni et al., 2020), obesity-associated meta-inflammation in metabolic organs, and diabetic patients hospitalized for COVID-19 (Foretz et al., 2019; Scheen, 2020). Metformin inhibits tumorigenesis possibly *via* mitochondrial respiratory chain complex 1. It also regulates neuronal oxidative stress, inflammation, and cell death to alleviate age-related neurodegenerative disease. Metformin might alleviate obesity-associated meta-inflammation through direct and indirect effects on various resident immune cells in metabolic organs. All these indicate the pleiotropic properties of metformin on multiple tissues through various underlying mechanisms. And the aforementioned mechanism receives intense debate for their contribution to metformin’s therapeutic effects (Foretz et al., 2014). In addition, various administration or drug delivery systems (e.g., microparticles or nanoparticles) are studied, besides traditional oral usage, which might help accelerate the development of novel therapeutic applications for this multifaceted drug (Foretz et al., 2019). This first-line agent has always been the focus of attention.

Currently, as the majority of the metformin publications are in-depth studies on molecular mechanisms and clinical application, most review articles on metformin have focused not only on specific but also relatively limited aspects like pharmacological activities or pharmacokinetics characteristics (Graham et al., 2011; McCreight et al., 2016). Thus, there is an increasing difficulty of carrying out new studies due to the insufficient integrated data or traditional reviews with limited quantification and repeatability. Bibliometrics analysis, a systematic analysis of publications assessing research status and trends, is thus used here to provide ideas and directions for future research.

Bibliometrics is a mathematical and statistical method, first used by Alan Pritchard in 1969 (Pritchard, 1969), which carries out retrospective reviews, looks for data correlations, makes predictions about future development, and so on (Thompson and Walker, 2015). Bibliometrics and visual analysis can efficiently support information integration to improve the understanding of the research activity. The purpose of this study was to build up essential, reliable, and thorough information on metformin in the last 40 years since its early emergence.

To achieve this, a bibliometrics analysis summarizing key information on authors, institutions, literatures, and their interrelationship from several databases were used. We hope to gain a deeper understanding on current hot areas and extend the current and strong discussion around the potential research directions.

## Materials and Methods

### Data Source and Search Strategy

The related publications from 1980 to 2019 were collected from the Science Citation Index Expanded (SCI-E) through the Web of Science (WOS) core database on April 6, 2020. With the search topic “metformin” and the literature types “article” and “review,” a total of 20, 526 articles satisfying these search criteria were pulled out to perform further analysis.

### Analytical Methods

HistCite 2.1, CiteSpace 5.5.R2, and VOSviewer version 1.6.14 were used in data analysis and data visualization. HistCite, developed by Eugene Garfield, is a powerful citation analysis software package to draw the development context of a certain research field and locate high impact literatures and academic masters (Garfield, 1987). CiteSpace, developed by Prof. Chaomei Chen, is a piece of software that visualizes networks among collaboration and documents citation as well as research hotspots (Chen, 2004; Chen, 2006). VOSviewer, developed by the Centre for Science and Technology Studies at Leiden University, is a piece of software focusing on bibliometrics networks such as co-citation, co-authorship, and key word co-occurrence. Altogether, these analytical tools provide objective and diverse views on the development of metformin research area.

## Results

### General Statistics

**FIGURE 1:**
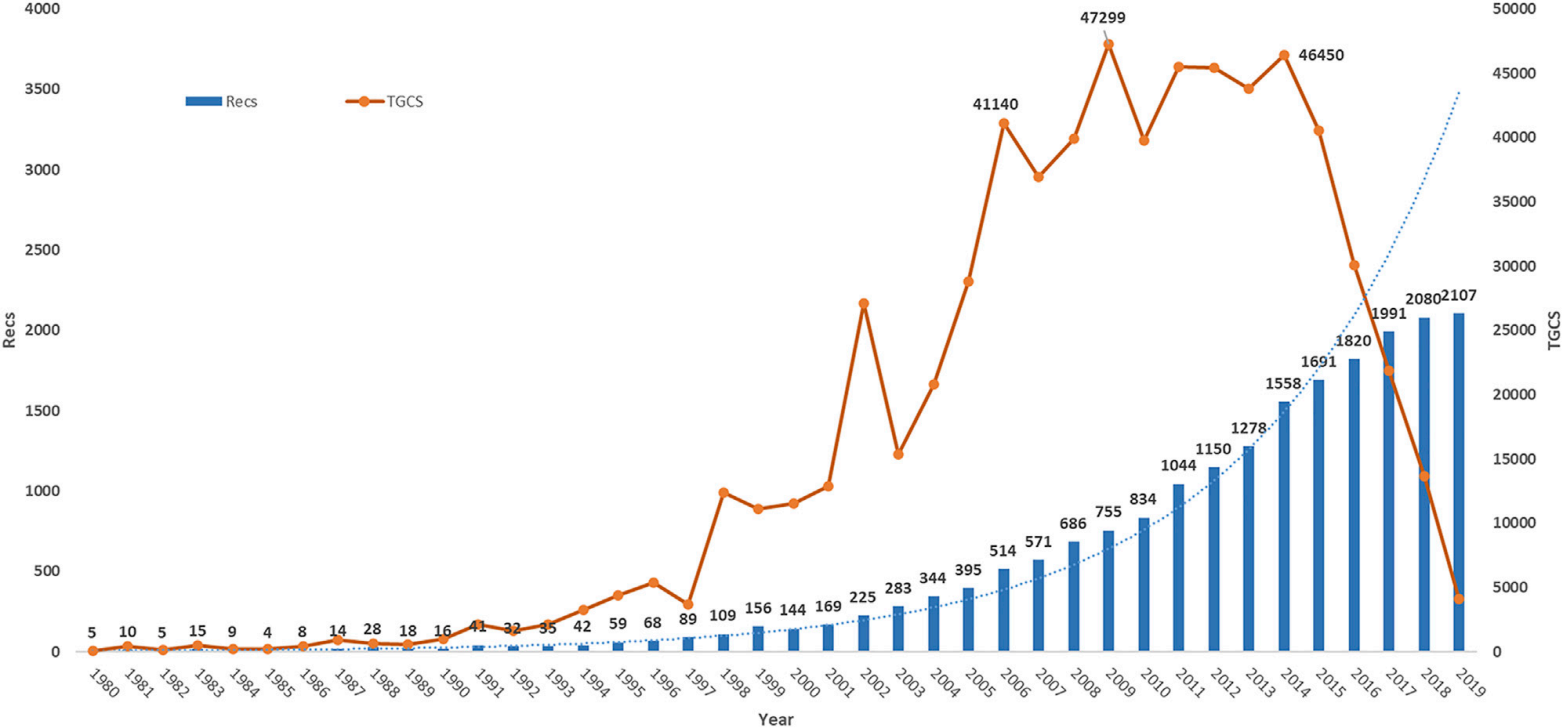
Timeline of publications and TGCS on metformin.

There are 20, 526 publications on metformin from the WOS core database during 1980–2019. We found that these articles were from 134 countries/regions, 15, 122 institutions, 69, 172 authors, and 2, 542 journals. They include 35, 212 keywords and were written in 19 languages. Majority of these articles were written in English (19, 948/20, 526), which account for 97.18%, and the next abundant ones were German, French, and Spanish, which include 226, 108, and 100 articles, respectively. There were two publication types: 17,479 records were research articles, which account for 85.16% and 3,047 records were reviews. As shown in Figure 1, the total number of records (Recs) increased steadily every year over the studied period. The total global citation score (TGCS) rapidly grew from 1990 to 2015, indicating the growing interests of metformin-related research. It is worth noting that TGCS reached the peak in 2009 with the highest value of 47, 299 articles, indicating there were significant findings in 2009. It then decreased in recent 5 years potentially due to the relative short period of time for the new articles to accumulate effects, despite the possibility of notable discoveries being found as well.

### Country/Region Characteristics

**FIGURE 2:**
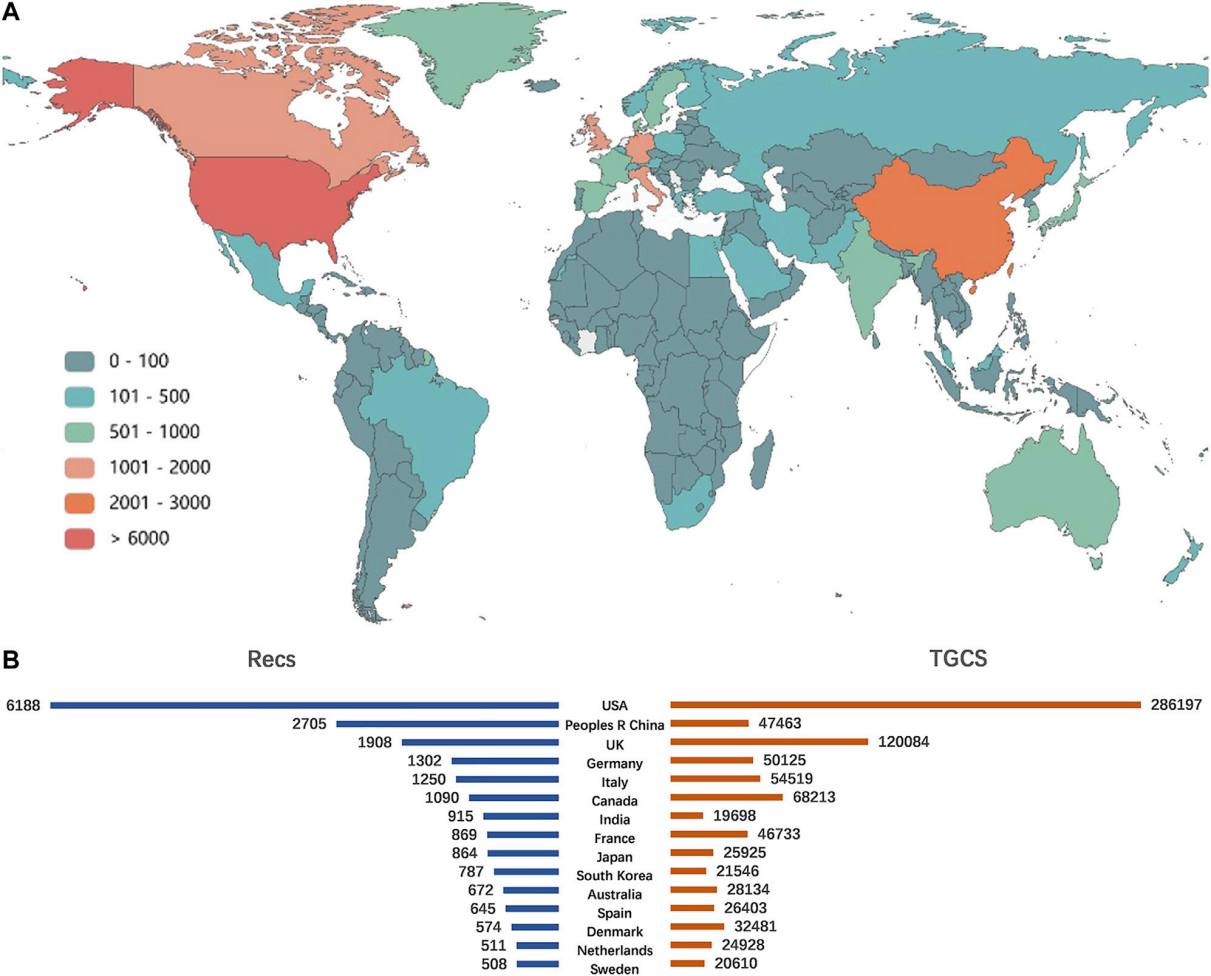
Main countries/regions distribution on metformin publications. **(A)** Geographical distribution of metformin. **(B)** The top 15 most productive countries in the publication of metformin and their corresponding TGCS.

During 1980–2019, groups from a total of 134 countries/regions published metformin-related articles. Figure 2 showed the global distribution of all the analyzed articles as well as the Recs and TGCS of the top 15 most productive countries. United States produced the most metformin-related articles with 6, 188 publications in the studied period. China, the United Kingdom, Germany, and Italy were the following countries in terms of the number of publications. The United States and the United Kingdom showed the highest TGCS of 286197 and 120084, respectively. Even though China is the second productive country, its TGCS ranked sixth with a TGCS of 47, 453, almost same as the TGCS of France which ranked eighth in number of publications.

### Academic Collaboration

**FIGURE 3:**
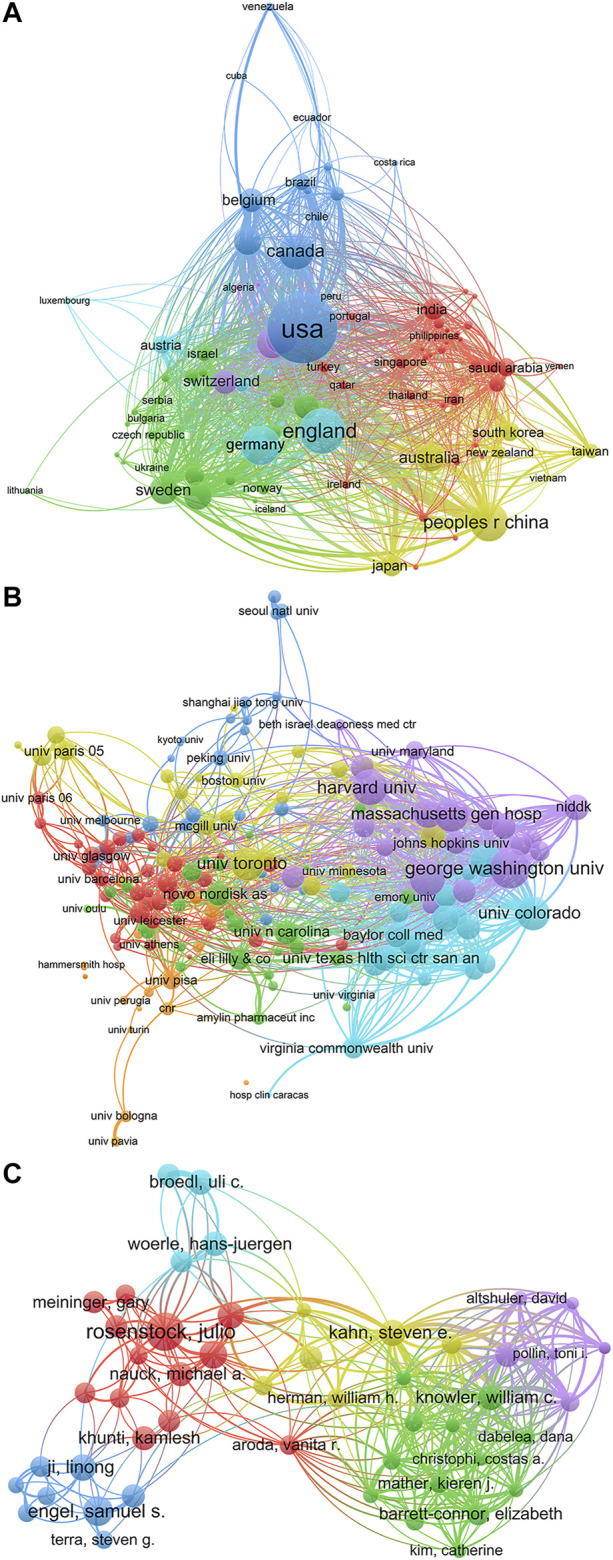
Academic collaboration between different countries/regions, institutions, and authors in metformin research area. **(A)** Academic collaboration between different countries/regions. **(B)** Collaboration between different institutions. **(C)** Collaboration between different authors.

Academic collaboration between different countries/regions, institutions, and authors usually greatly facilitates knowledge and reagents exchange which broadens the vision of the field. As expected in metformin research field, notable cooperative relationships happen at multiple levels (Figure 3). The cooperation network visualization was performed by VOSviewer. In Figure 3A, each circle/node represents a different country/region, the size of the circle/node represents the number of published documents, the thickness of connecting lines represents the strength of cooperation with each other, and each color represents a cluster—a set of items with similar attributes within a network. Ninety-two countries/regions meet the threshold of a minimum of five documented collaboration. The United States, the United Kingdom, and Germany had the highest number of collaborations with other countries/regions. Interestingly, countries within the top 10 of publication numbers (Figure 2B) also have high collaboration with others, except for India, Japan, and South Korea.

Another informative way to evaluate the status of collaboration is to look at the partnership between institutions. With the threshold of total citations of more than 2, 000 times, 174 institutions were selected and drawn in Figure 3B. Again, each circle represents a certain institution, the thickness of connecting lines indicates the cooperation intensity, and each color represents a cluster. If we take a closer look at the top 10 most collaborative institution list (Table 1), three pharmaceutical companies, Eli lilly & Co., AstraZeneca, and Novo Nordisk A/S take up three places, suggesting a tight communication between basic research studies and clinical application and manufacturing of metformin, which is not surprising as it is one of the most prescribed first-line medication to control blood glucose. And one hospital, Massachusetts General Hospital, which is one of the oldest and largest affiliated teaching hospitals of Harvard University, ranked third in this list. All other institutes are high ranking research universities in the world.

**TABLE 1:** The top 10 most collaborative institution.

Organization	Document	Citation	Total collaborative strength
Univ Toronto	268	24,825	191
Harvard Univ	246	22,774	184
Massachusetts Gen Hosp	143	10,911	134
Eli Lilly & Co.	214	14,366	129
George Washington Univ	152	22,661	129
Univ Washington	176	10218	119
Astrazeneca	172	5,187	108
Novo Nordisk A/S	174	11,877	103
Univ Calif San Diego	172	9,312	97
Univ Colorado	143	6,926	96

To gain more insight into the research status within specific research groups, the co-authorship analysis was performed on the 69, 172 authors. A fractional counting method was used to reduce the influence of documents with many authors. The clustering analysis is a data mining technology that can obtain several structured clusters to discover the topic distribution and organizational structure in the knowledge domain (Lin et al., 2019). When cluster analysis was performed on the top 100 authors with the most publications, 55 authors were found to form clusters with each other (Figure 3C). The tight interweaving intersections between these author clusters suggest that the scientists all around the world have a close cooperation network. It is worth mentioning that Julio Rosenstock, Samuel S. Engel, and Knowler William C. are the key nodes of the collaboration network.

### Funding Agency Distribution

Research funding agencies significantly affect the research subjects and directions. Table 2 listed the top 10 agencies that provided the most funds to metformin-related research from 1980 to 2019, which accounts for 42.7% of all the publications. Notably, consistent with their high publication, citation, and collaboration level, three United States research funding agencies sponsored 4, 291 articles, which accounted for one-fifth of all the publications (20.9%). Meanwhile, six international pharmaceutical companies were on this list with 3, 311 published articles accounting for 16.13%. These mostly include studies driven by optimization of clinical usage like optimizing dosage forms, drug combination, improving efficacy, and so on. The National Natural Science Foundation of China sponsored 1, 164 articles and ranked third, which is also refracted by the high numbers of publication from groups in China.

**TABLE 2:** The top 10 productive funding agencies in metformin area.

Institution	Recs (#)	Percentage (%)
United States Department of Health Human Services	1,930	9.40
National Institutes of Health NIH, United States	1,888	9.20
National Natural Science Foundation of China	1,164	5.67
Novo Nordisk	721	3.51
Astrazeneca	625	3.05
Eli Lilly& Co.	602	2.93
Bristol Myers Squibb	478	2.33
NIH National Institute of Diabetes Digestive Kidney Diseases (NIDDK)	473	2.30
Merck & Co.	452	2.20
Boehringer Ingelheim	433	2.11

## Institution and Author Contributions

A total of 15, 122 institutions and 69, 172 authors have contributed to the field of metformin. Table 3 showed the top 15 institutions with the highest number of Recs or TGCS, respectively. Notably, University of Toronto occupied the first place in both Recs and TGCS ranks. Although University of Dundee and Yale University do not rank the top 10 with their Recs number, they have quite high TGCS rankings showing their high impact contributions.

**TABLE 3:** The top 15 productive institutions in terms of Recs and TGCS in metformin area.

Institution	Recs	Institution	TGCS
Univ Toronto (Canada)	273	Univ Toronto (Canada)	28672
Harvard Univ (United States)	248	George Washington Univ (United States)	27998
Eli Lilly & Co. (United States)	214	Harvard Univ (United States)	22893
Univ Copenhagen (United States)	190	Univ Dundee (United Kingdom)	16290
Univ Washington (United States)	182	Yale Univ (United States)	14644
Seoul Natl Univ (South Korea)	178	Eli Lilly & Co. (United States)	14366
Novo Nordisk A/S (Denmark)	176	McGill Univ (Canada)	13539
Univ Calif San Diego (United States)	176	Massachusetts Gen Hosp (United States)	13244
AstraZeneca (United Kingdom)	172	Univ Washington (United States)	12505
Shanghai Jiao Tong Univ (China)	169	Univ N Carolina (United States)	12458
McGill Univ (Canada)	164	Novo Nordisk as (Denmark)	11904
George Washington Univ (United States)	157	Univ Calif San Diego (United States)	11586
Massachusetts Gen Hosp (United States)	148	Univ Texas (United States)	11090
Univ Colorado (United States)	147	Univ Pittsburgh (United States)	9641
Peking Univ (China)	146	Boehringer Ingelheim Pharma GmbH & Co. KG (Germany)	9633

**FIGURE 4:**
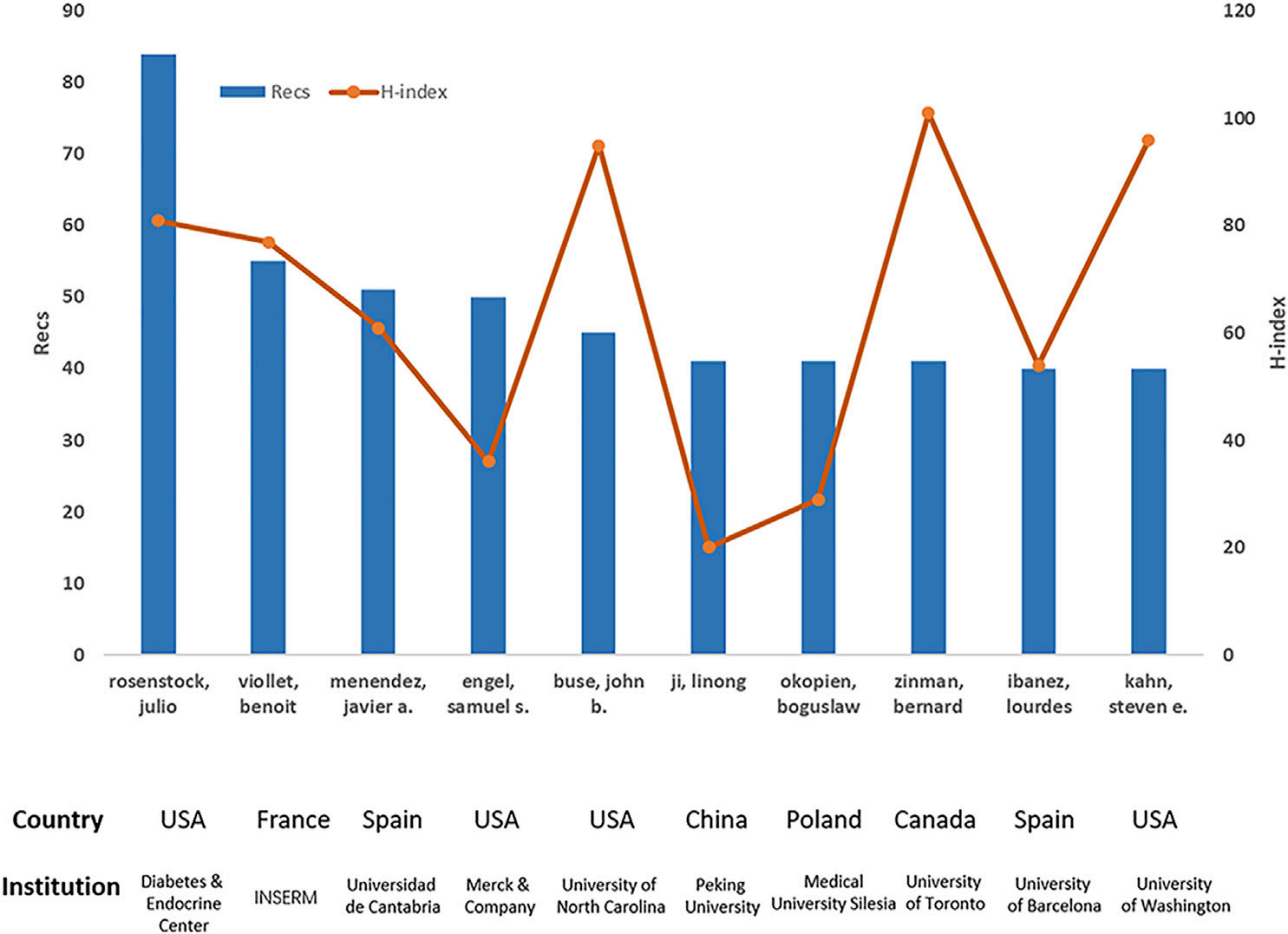
The top 10 most productive authors in metformin research area.

To discover the most impactful experts in the metformin field in the last 4 decades, all the authors were ranked based on their publication number and an H-index. The H-index was first introduced by Hirsch in 2005. It is a comprehensively calculated score to evaluate the importance, significance, and broad impact of a scientist’s cumulative research contributions (Hirsch, 2005). Figure 4 depicted the top 10 most productive authors with their corresponding H-index. Julio Rosenstock from Diabetes and Endocrine Center of United States is the most productive author with 84 records and an H-index of 81, owning 40 highly cited articles with a TGCS of more than 100. The highest H-index owner is Bernard Zinman from University of Toronto with Recs of 41. John B. Buse from University of North Carolina and Steven E. Kahn from University of Washington had an H-index of 95 and 96, respectively.

### Journal Performance

The 20, 526 publications analyzed were published in 2, 542 journals. The top 10 journals with the most publications account for 16.15% of all the articles (Table 4). Four journals were from the United States, three journals were from the United Kingdom, and others were from the Netherlands, Germany, and France. Impact factor (IF) is a putative marker of journals’ influence based on the frequency of a journal’s articles cited by other scientific publications (Garfield, 1999). In comparison, TLCS represents the number of citations from local publications which are strongly associated with closely related fields. Therefore, an article with high TLCS suggests its pioneering position in the corresponding research area. During the rapid development period of metformin research (1999–2019, Figure 1), the journal Diabetes, Obesity and Metabolism had the most abundant publications (Recs of 760). The top three journals with the highest IF (2018) are Diabetes care, *Diabetologia,* and Journal of clinical endocrinology and metabolism.

**TABLE 4:** The top 10 journals ranked by their Recs in metformin area.

Rank	Journal	Country	Recs	TLCS	TGCS	IF (2018)	H-index	ISSN
1	Diabetes Obesity and Metabolism	United Kingdom	760	11137	27288	6.133	112	1462-8902
2	Diabetes Care	United States	546	21177	54246	15.27	328	0149-5992
3	PLos One	United States	390	0	8885	2.776	268	1932-6203
4	Diabetes Research and Clinical Practice	Netherlands	296	1970	5652	3.239	100	0168-8227
5	Diabetic Medicine	United Kingdom	281	4327	11888	3.107	132	0742-3071
6	Journal of Clinical Endocrinology and Metabolism	United States	279	7115	21176	5.605	328	0021-972x
7	Diabetologia	Germany	205	4876	15706	7.113	207	0012-186x
8	Oncotarget	United States	191	1355	3875	0	91	1949-2553
9	Current Medical Research and Opinion	United Kingdom	187	2111	5500	2.345	98	0300-7995
10	Diabetes and Metabolism	France	180	2023	4380	4.008	79	1262-3636

### Category Analysis

**FIGURE 5:**
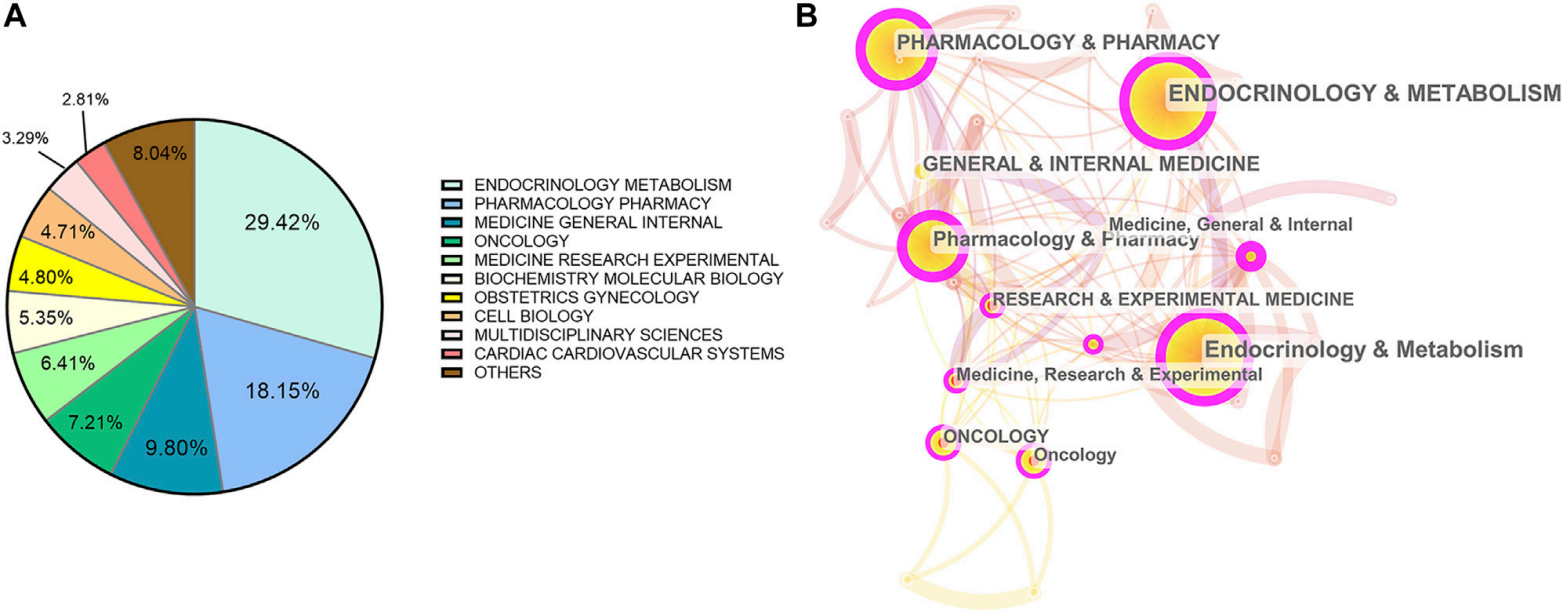
Analysis of categories. **(A)** The top 10 most published subjects within metformin area. **(B)** A category visualization of metformin research.

Next, we sought to collect the most published subject categories within metformin research field among all the publication. Figure 5A illustrates the top 10 categories of studies that are mostly published and the proportion of their corresponding articles. The top three categories account for nearly 60% of all the publications, which include endocrinology metabolism with 6, 039 records (29.42%), pharmacology/pharmacy with 3, 725 records (18.15%), and general internal medicine with 2, 013 records (9.81%).Figure 5B presents a category visualization analysis of metformin research within the past 40°years by CiteSpace. The categories of top 10 most cited articles from each year were analyzed and the categories cited by more than 1,000 times were selected. Each node represents a certain category and its size represents its frequency of occurrence. The lines represent their interrelation. Centrality is an index to evaluate the importance of a node in a network and purple circles mark nodes with high centrality (Figure 5B). Red dots in the center represent burst categories, which refer to the sudden increase in the number of publications within the recent years, reflecting the research frontier and development trend of the field (Chen and Morris, 2003). We found that endocrinology metabolism and pharmacology/pharmacy have always been important in the field, while oncology, medicine and experimental medicine have the strongest citation burst, suggesting quick evolving research areas in near future.

### Keyword Detection and Burst Analysis

**FIGURE 6:**
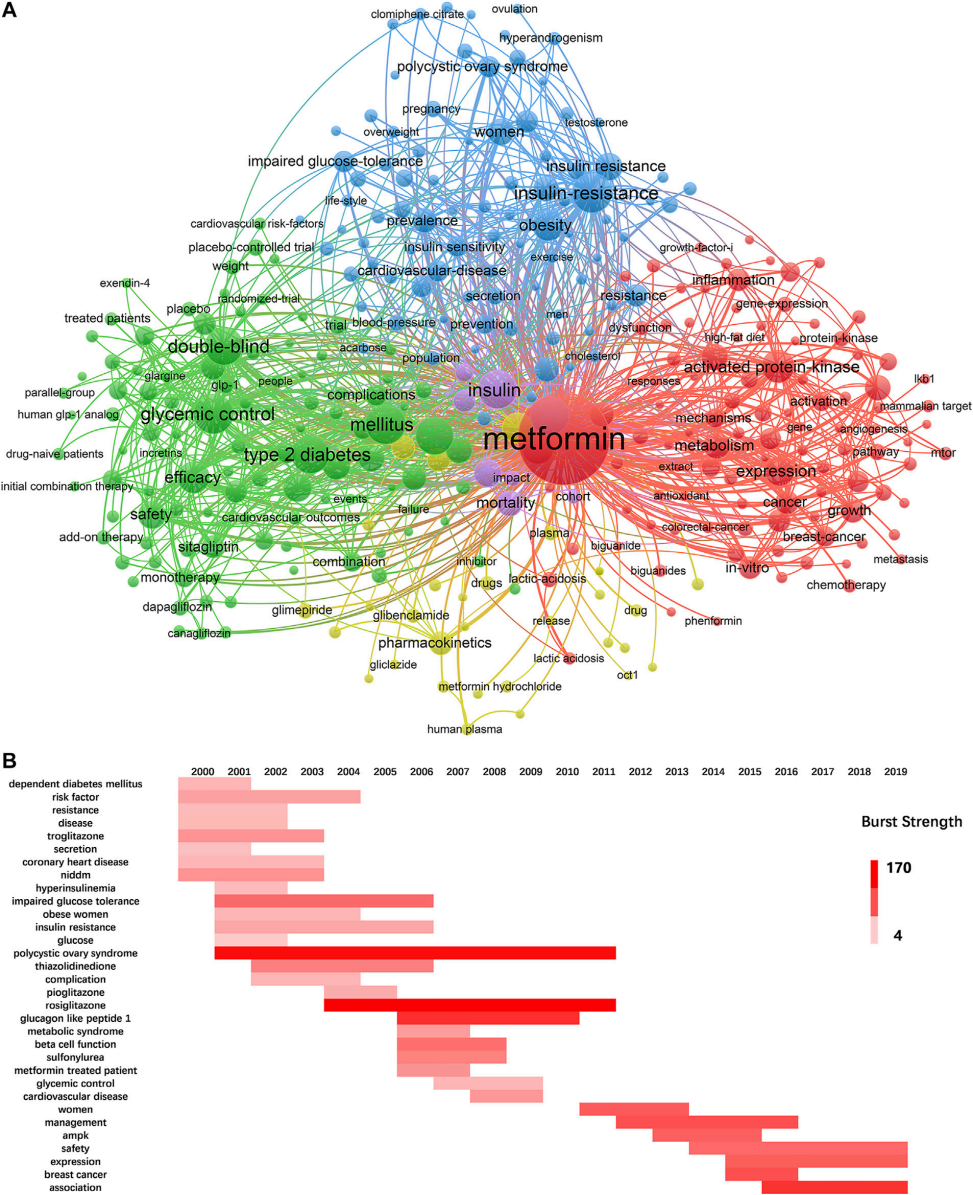
Keywords analysis. **(A)** The cluster analysis of keywords with an occurrence more than 100 in metformin area, 1980–2019. **(B)** The burst detection of keywords of metformin area in a chronological order, 2000–2019.

The keywords can reflect the frontiers and hotspots in a specific field. The top 10 keywords with the highest occurrence frequencies were metformin (11, 338), mellitus (2, 217), glycemic control (2, 202), insulin-resistance (2, 167), insulin (2, 076), risk (2, 008), therapy (1, 982), type 2 diabetes (1, 954), double-blind (1, 919), and activated protein-kinase (1, 460). A cluster visualization of keywords was performed with VOSviewer. With a cutoff of occurrence > 100, 334 keywords were selected and five clusters emerged by co-occurrence clustering analysis. As shown in Figure 6A, each circle represents one of the 334 keywords and its size represents the frequency of occurrence. The connecting line indicates that the two keywords connected to it have at least one co-occurrence. The five clusters represented by different colors are as follows: 1) Mechanism in red: activated protein-kinase, AMPK expression, inhibition, metabolism, and oxidative stress; 2) Clinical trials in green: association, combination, dipeptidyl peptidase-4 inhibitor, double-blind, mellitus, safety, and therapy; 3) Diseases in blue: cardiovascular disease, insulin-resistance, metabolic syndrome, obesity, and polycystic ovary syndrome; 4) Pharmacokinetics in yellow; and 5) other low quantity key words in purple, such as mortality, insulin, and risk.

To unravel the fastest growing topics in recent years, the keywords in top 20 most cited articles within the last 2 decades were selected to perform a burst analysis in one-year slice (Figure 6B). The deeper the color the more popular the research area is. While some keywords have a burst for only 1–2 years, rosiglitazone, polycystic ovary syndrome, glucagon-like peptide-1, association, and breast cancer have occupied the top five positions of highest burst strength and lasted for multiple years, suggesting continuous research interests and focus. The most recent hotspots were association, expression, and safety.

### Co-Citation Analysis and Roadmap of Metformin Development

**FIGURE 7:**
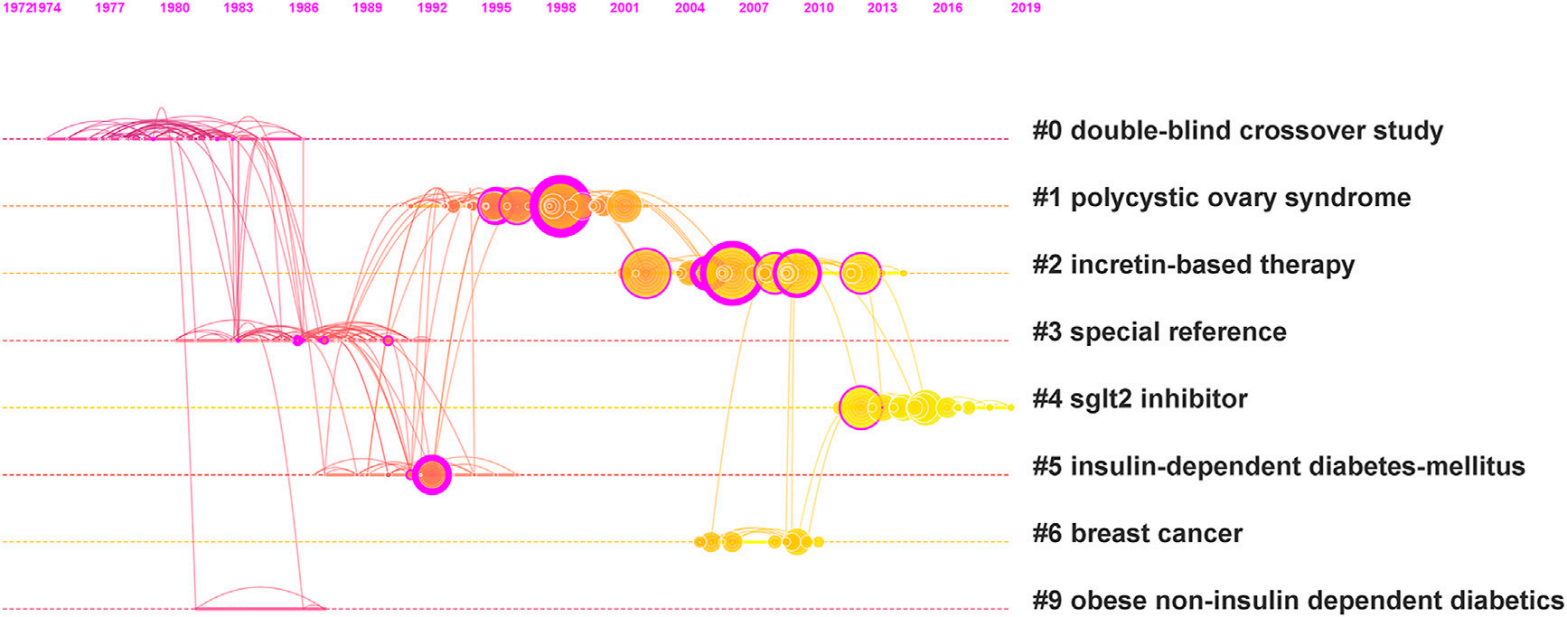
Visualized timeline of co-citation clusters in metformin research area.

When two or more articles are cited by > 1 later publication at the same time, they constitute a co-citation relationship. Higher co-citation number represents stronger co-citation relationship, which thus indicates the high similarity of these articles and common themes will emerge from these articles. To further delineate the research frontiers and obtain critically cited publications from a chronological standpoint, we performed a cluster analysis from a co-citation relationship analysis by CiteSpace. Figure 7 demonstrated a visualized timeline of the top 10 most cited articles of metformin each year based on the co-citation analysis. There are 190 nodes and the lines connecting different circles represent the co-citation relationship. Eight cluster tags representing hot common themes were selected and ranked on the right of Figure 7 based on the log-likelihood ratio algorithm. Along the dashed line linked to each tag, circles with larger radius represent higher citation frequencies and warmer colored lines represent later publication dates.

The more detailed information of these clusters was listed in Table 5. Two parameters, modularity Q and silhouette value were calculated to evaluate the structure significance and robustness of these clusters, respectively. A cluster structure with the modularity Q higher than 0.3 was considered significant, and with the modularity Q of 0.7025 (Figure 7), ours have cluster structure with high significance. A silhouette value greater than 0.7 was considered convincing (Chen, 2004), and all of our clusters are convincing.

**TABLE 5:** The detailed information of the 8 clusters in Figure 7.

Cluster Id	Size	Silhouette	Mean year	Cluster label
0	28	0.868	1979	Double-blind crossover study
1	32	0.863	1996	Polycystic ovary syndrome
2	30	0.936	2006	Incretin-based therapy
3	29	0.773	1987	Special reference
4	18	0.93	2015	Sglt 2 inhibitor
5	13	0.843	1991	Insulin-dependent diabetes mellitus
6	10	0.956	2007	Breast cancer
9	3	0.916	1984	Obese non-insulin-dependent diabetes

Just like most of the other research fields, the hot common themes in metformin research area have changed over the years (Figure 7). Specifically, in the 1980s, double-blind crossover studies were performed widely to verify the efficacy of treating obese and non-insulin–dependent diabetes. Special references, that is, studies on metformin in addition to the treatment of diabetes also emerged. In the 1990s, polycystic ovary syndrome and insulin-dependent diabetes mellitus had become hotspots. In 2006 and 2007, incretin-based therapy and breast cancer had attracted increasing attention. The latest research trend (2015) is about sodium-dependent glucose transporters 2 (SGLT2) inhibitor, which was considered a new breakthrough of treating diabetes.

To paint a bigger picture of how certain milestones, that is, critical events/publications contribute to the overall metformin development over the studied period, HisCite was used to collect the top 15 articles with the highest TLCS (ranging from 286 to 1709) (Table 6)

**TABLE 6:** The top 15 most cited articles.

Authors	Journal	IF (2018)	H-index	Title	TLCS	TGCS
Turner RC et al.	Lancet	59.102	700	Effect of intensive blood glucose control with metformin on complications in overweight patients with type 2 diabetes (UKPDS 34)	1709	5207
Zhou GC et al.	Journal of Clinical Investigation	12.282	447	Role of AMP-activated protein kinase in mechanism of metformin action	1625	3470
Knowler WC et al.	New England Journal of Medicine	70.67	993	Reduction in the incidence of type 2 diabetes with lifestyle intervention or metformin	1161	10,538
Bailey CJ et al.	New England Journal of Medicine	70.67	993	Drug therapy–Metformin	930	1406
Evans JMM et al.	BMJ-British Medical Journal	27.604		Metformin and reduced risk of cancer in diabetic patients	908	1389
Kahn SE et al.	New England Journal of Medicine	70.67	993	Glycemic durability of rosiglitazone, metformin, or glyburide monotherapy	813	1991
Owen MR et al.	Biochemical Journal	4.331	243	Evidence that metformin exerts its anti-diabetic effects through inhibition of complex 1 of the mitochondrial respiratory chain	761	1209
Turner RC et al.	JAMA-Journal of the American Medical Association	51.273	622	Glycemic control with diet, sulfonylurea, metformin, or insulin in patients with type 2 diabetes mellitus—Progressive requirement for multiple therapies (UKPDS 49)	745	1676
Nathan DM et al.	Diabetes Care	15.27	328	Medical Management of Hyperglycemia in Type 2 Diabetes: A Consensus Algorithm for the Initiation and Adjustment of Therapy A consensus statement of the American Diabetes Association and the European Association for the Study of Diabetes	714	2101
Holman RR et al.	New England Journal of Medicine	70.67	993	10-year follow-up of intensive glucose control in type 2 diabetes	691	4080
Shaw RJ et al.	Science	41.037	1058	The kinase LKB1 mediates glucose homeostasis in liver and therapeutic effects of metformin	607	1250
Defronzo ra et al.	New England Journal of Medicine	70.67	993	Efficacy of metformin in patients with non-insulin-dependent diabetes-mellitus	579	896
Stumvoll m et al.	New England Journal of Medicine	70.67	993	Metabolic effects of metformin in non-insulin-dependent diabetes mellitus	556	807
Viollet B et al.	Clinical Science	5.237	126	Cellular and molecular mechanisms of metformin: an overview	542	823
Zakikhani M et al.	Cancer Research	8.378	411	Metformin is an AMP kinase–dependent growth inhibitor for breast cancer cells	526	734

There were many notable events in the history of metformin development. Metformin was first discovered in *Galega officinalis* in Europe for its efficacy to reduce blood glucose in 1918 (Sanchez-Rangel and Inzucchi, 2017), but phenformin and buformin, other two members from the same biguanide group, had been withdrawn due to their side effect of lactic acidosis reported in most countries in 1977–1980 (Nattrass and Alberti, 1978). After more rigorous safety and efficacy research, metformin was approved in 1994 and introduced in 1995 in the United States (Bailey, 2017). With the deeper understanding of the mechanisms, International Diabetes Federation (IDF) recommended metformin as an initial glucose-lowering pharmacotherapy for type 2 diabetes in 2005 (International Diabetes Federation Guideline Development Group, 2014). In 2008, with a 10-year follow-up study, metformin was also found to reduce cardiovascular risk continually (Holman et al., 2008). In 2011, metformin was included in the WHO’s essential medicines list (World Health Organization, 2019). All the processes are closely related to studies on the mechanisms of hypoglycemic effect of metformin. One main mechanism is through decreasing hepatic glucose production, by inhibiting hepatic gluconeogenesis through liver kinase B1 (LKB1)/adenosine monophosphate–activated protein kinase (AMPK) pathway, and AMPK-independent inhibition of mammalian target of rapamycin1 (mTORC1), which subsequently inhibit mitochondrial glycerophosphate dehydrogenase (Musi et al., 2002; Shaw et al., 2005; Kalender et al., 2010; Madiraju et al., 2014). In addition, metformin can also increase glucose utilization by the peripheral tissues through SRC homology two domain–containing inositol-5-phosphatase 2 (SHIP2) pathways (Shaw et al., 2005). In terms of clinical application, the incidence of lactic acidosis is very low in patients treated with metformin owning to that the oral absorption, hepatic uptake, and renal excretion of metformin are mediated very largely by organic cation transporters (OCTs) (Graham et al., 2011). Therefore, maintaining reasonable plasma concentrations of metformin can accomplish efficacy without sacrificing safety.

Other relatively new hypoglycemic drug types were also fast-growing research focuses. For example, glucagon-like peptide-1 (GLP-1) is a gut-derived incretin hormone that can suppress glucagon secretion, stimulate insulin secretion, inhibit gastric emptying, and reduces appetite and food intake (Drucker and Nauck, 2006). Several drugs functioning through this pathway were currently used in clinic or in late phase clinical trials. Liraglutide, a once-daily human GLP-1 analogue, is a popular treatment option for type 2 diabetes, especially considering its co-effect on weight loss and reduced risk of hypoglycemia (Buse et al., 2009; Garber et al., 2009). Dipeptidyl peptidase-4(DPP-4) inhibitors can inhibit the degradation of GLP-1 and protect function of insulin-secreting β cells. Exenatide and sitagliptin, two DPP-4 inhibitors, were efficacious and well tolerated in individuals with type 2 diabetes combined with the treatment of metformin (DeFronzo et al., 2005; Charbonnel et al., 2006). What is more is that peroxisome proliferators–activated receptors γ (PPARγ) agonist rosiglitazone, SGLT2 inhibitor empagliflozin, and glimepiride from sulfonylurea family have also been confirmed to have stable efficacy and are well tolerated (Fryer et al., 2002; Nissen and Wolski, 2007; Zinman et al., 2015). All in all, combined treatment of metformin with one or more of these newer agents have been reported to treat type 2 diabetes more efficiently (Fryer et al., 2002; DeFronzo et al., 2005; Buse et al., 2009; Zinman et al., 2015).

Notably, metformin also gains growing popularity in basic research and clinical application to treat polycystic ovary syndrome (PCOS) and breast cancer (Figure 7)since 1990s. Studies have shown that metformin can reduce hyperinsulinemia and hyperandrogenemia, and improve in menstrual abnormalities and resumption of ovulation in women with PCOS (Moghetti et al., 2000). Metformin was also associated with a decreased risk of cancer incidence compared with other blood glucose controlling treatments among diabetic patients (Decensi et al., 2010). The effects and potential mechanisms of metformin in treating PCOS and breast cancer are listed in Table 7. In all, AMPK-related pathways play a crucial role in the treatment of diabetes and other diseases with metformin.

**TABLE 7:** The effects and potential mechanisms of metformin on PCOS and breast cancer.

Diseases	Mechanisms	References
PCOS	Decreasing Insulin Resistance and Hyperinsulinemia	Morley et al. (2017)
	Inhibiting the Production of Steroid Hormones by Ovarian Cells	Mansfield et al. (2003); Tosca et al. (2007); Faure et al. (2018)
	Stimulating AMPK and the Triggering of AMPK-Dependent Pathways in Ovarian Cells	Attia, Rainey and Carr. (2001); Tosca et al. (2005), (2007); Faure et al. (2018)
Breast cancer	Activating AMPK-Independent Pathways and then Inhibiting IGF-1/IGF-1R, PI3K-, and MAPK-Signaling Pathways	Heckman-Stoddard et al. (2016)
	Activating AMPK-Dependent Pathways; Regulated by Metformin Suppression Inhibition of Mitochondrial Complex-I	Shaw et al. (2005b); Madiraju et al. (2014b); Sekino et al. (2018); Mishra and Dingli, (2019)
	Others: Acting on the Tumor Microenvironment and Acting on Endothelial Cells.	Wang et al. (2015); Kurelac et al. (2020)

### Emerging New Research Frontiers

Considering the time needed for the more recent publications to accumulate influence, major research advances could have been missed using TLCS alone to evaluate the importance of a publication. Therefore, we chose articles published at high H-index (>600) journals as the secondary criterion to analyze the articles from 2015 to 2019 separately. A total of 39 records were pulled out from the journals Nature (IF = 43.07, H-index = 1096), Science (IF = 41.037, H-index = 1085), Cell (IF = 36.216, H-index = 705), LANCET (IF = 59.102, H-index = 700), New England Journal of Medicine (IF = 70.67, H-index = 933), and Journal of the American Medical Association (IF = 51.273, H-index = 622). Indeed, several interesting discoveries were found in these records. For example, The effect of metformin treating type 2 diabetes could function through altering gut microbiome composition and function (Forslund et al., 2015; Pryor et al., 2019); a novel mechanism of metformin lowering body weight by elevating circulating levels of growth and differentiation factor 15(GDF15) (Coll et al., 2020); anti-aging activity of metformin (Castillo-Quan and Blackwell, 2016); and in endometrial cancer research area, metformin reduces cellular proliferation by inhibition of the PI3K-AKT-mTOR pathway in preclinical studies (Sivalingam et al., 2015).

## Discussion

We carried out a bibliometrics analysis on 20, 526 metformin articles from the WOS core collection database during 1980–2019 using multiple literature analysis software and computational algorithm. This work summarized research topics, trends, and sources for metformin as well as obtained an outline of global research on its impact.

During the studied time period, the number of publications every year continued to grow indicating the fast development and continuous research interests of metformin. The TGCS suddenly increased quickly at 1998, which was associated with one important RCT study of UKPDS recommending metformin as first-line drug for diabetes (UK Prospective Diabetes Study (UKPDS) Group, 1998). This trial was considered the most fundamental and an important study and greatly advanced this research field. In 2002, another clinical study showed that metformin treatment reduced the incidence of diabetes in people at high risk (Knowler et al., 2002). This continued to strongly drive the annual output. In 2006, the repurposing of metformin for cancer (Zakikhani et al., 2006), the clinical evaluation for diabetes (Kahn et al., 2006), and the potential molecular mechanism (Shaw et al., 2005) further promoted the development of this field in next few years.

As for the country, the United States is the absolute leader who has the most publication and highest academic reputation overall. This could be due to pioneer research institutions like the George Washington University, Harvard University, Yale University, and Massachusetts General Hospital, a top hospital contributing to a great number of clinical research studies. It was worth mentioning that three of the most influential authors/groups, Julio Rosenstock, Samuel S. Engel, and John B. Buse, were also from institutes of the United States. Although other countries, namely China, the United Kingdom, Germany, Italy, and Canada, had relatively low Recs, their considerable progress drives recent increases in their annual output, which is consistent with strong collaborations among them. A multicenter, prospective, randomized intervention trial of diabetes was done by the United Kingdom Prospective Diabetes Study (UKPDS) during 1977–1997, which contributed a series of important results until now. Therefore, the United Kingdom had the second TGCS as the third driving force for Recs. Besides the United Kingdom, many European countries (Germany, Italy, and France) had a relatively high influence that outweighed their publication number. This may be related to the long history of using *Galega officinalis* in Europe. Notably, University of Toronto had a great impact in metformin field that ranked top one in both Recs and TGCS among all the institutes. This contributed to the third TGCS of Canada and indicated Canada as a potential important influence on future developments. The metformin research in China developed rapidly in recent years, with the first well-documented record in 1998. This development opportunity may be related to the fact that Glucophage (metformin hydrochloride tablets) from Bristol Myers Squibb came into the market in 1999 and it has dominated Chinese market ever since. The publication from Chinese groups has ranked second in quantity, but the research studies should be extended both in the breadth and depth to improve the international influence.

In terms of scientific collaboration, the United States, the United Kingdom, and Germany had the most corporations; Institute-wise, University of Toronto, Harvard University, and Massachusetts General Hospital had the most corporations; Author-wise, Julio Rosenstock appears to have the widest collaborative network. Bernard Zinman from University of Toronto, Steven E. Kahn from University of Washington, and John B. Buse from University of North Carolina are the most influential based on their highest H-index. They also had an active collaboration, which maximized the regional advantages and further strengthening their academic impact.

It is worth mentioning that large multinational pharmaceutical companies play a key role in modulating the basic research directions. For example, combined therapy to treat type 2 diabetes is the most current drug development direction, products representing this trend include Synjardy (empagliflozin and metformin hydrochloride) and Jentadueto (linagliptin and metformin hydrochloride) produced by Eli Lilly & Co. and PrandiMet (repaglinide and metformin hydrochloride) produced by Novo Nordisk AS, and the application method is moving toward sustained release preparation.

The most studied mechanisms of metformin in modulating blood glucose and exerting other metabolic benefits are AMPK-dependent and AMPK-independent pathways to inhibit mitochondrial respiration, glycerophosphate dehydrogenase, and a mechanism involving the lysosome (Rena et al., 2017). In addition, new application of metformin and drug–drug interaction has been studied widely in the last 20 years. First, the hypoglycemic mechanism of metformin may involve regulating gut microbiome (Forslund et al., 2015), which should be further studied. Second, as a conventional drug, metformin can also be used in treating obesity, polycystic ovary syndrome, cancer (especially breast cancer), and delaying aging (Moghetti et al., 2000; Dowling et al., 2007; Coll et al., 2020). Third, GLP-1 analogue like liraglutide, DPP-4 inhibitor like exenatide and sitagliptin, SGLT two inhibitor like empagliflozin, and PPARγ agonist like rosiglitazone are new anti-diabetes agents under development. Using them combined with metformin may provide better results (Fryer et al., 2002; DeFronzo et al., 2005; Charbonnel et al., 2006; Garber et al., 2009; Zinman et al., 2015). The major side effect of metformin was its gastrointestinal intolerance. The most common gastrointestinal manifestations were abdominal pain, nausea, diarrhea, and vomiting, which caused 1.2–5% of all patients stopping therapy (Bouchoucha et al., 2011). The metformin gastrointestinal intolerance was closely related to the genetic variations of organic cation transporter 1 (OCT1), which played a role in the hepatic uptake of metformin and OCT1 was also proved to affect metformin therapeutic action (Shu et al., 2007). Clinical pharmacokinetics of metformin was studied to minimize the development of the adverse effect (Graham et al., 2011). The great improvement to overcome side effects was the gradual release of immediate-release metformin or the use of extended-release and delayed-release formulations of the drug (Bonnet and Scheen, 2017). In addition, emerging evidence suggested that metformin could improve meta-inflammation in metabolic organs *via* direct and indirect effects on tissue-resident immune cells, which also contributed to diabetic patients hospitalized for COVID-19 (Foretz et al., 2019; Scheen, 2020).

As the hot research subject categories evolved in metformin field throughout the time, endocrinology metabolism and pharmacology/pharmacy are the long-standing main research focus, while oncology, medicine and experimental medicine are the emerging burst categories. Through keywords cluster analysis, we found that the metformin research mainly focused on its efficacy and safety in treating type 2 diabetes. This gave the most frequent keywords that include glycemic control, insulin-resistance, risk, and activated protein-kinase. By keyword burst analysis and co-citation analysis, the hot topics throughout the years are rosiglitazone, polycystic ovary syndrome, glucagon-like peptide-1, association, breast cancer, incretin-based therapy, and SGLT 2 inhibitor. The combination of aforementioned analysis showed three main trends: 1) Clinical studies to evaluate metformin at the individual level in terms of glycemic control, cardiovascular outcome, and mono/combined therapy. This promoted a better understanding of its clinical efficacy might and helped clinicians develop a personalized approach in metformin therapy. Meanwhile, pharmacokinetical or controlled-release research has already paved the way. 2) Clinical or research studies to evaluate improvements in obesity-associated and tissue-specific insulin sensitivity through direct and indirect effects in metabolic organs. 3) The repurposing of metformin for further applications on cancer, age-related, and metabolism diseases, highlighting its vast range of different possible actions.

## Conclusion

In this review, this bibliometrics analysis study provides a systematic view of the evolutionary process, research hotspots, and future directions of metformin research, drug development, and clinical application. It will help both long-time experts and younger generation researchers who are just entering the metformin field to better understand the global research status and trends.

## References

[B4] Aljofan M. Riethmacher D. (2019). Anticancer Activity of Metformin: A Systematic Review of the Literature. Future Sci. OA 5 (8), Fso410. 10.2144/fsoa-2019-0053 31534778 PMC6745597

[B43] UK Prospective Diabetes Study (UKPDS) Group (1998). Effect of Intensive Blood-Glucose Control With Metformin on Complications in Overweight Patients With Type 2 Diabetes (UKPDS 34). Lancet 352 (9131), 854–865. 9742977

[B21] Bailey C. J. (2017). Metformin: Historical Overview. Diabetologia 60 (9), 1566–1576. 10.1007/s00125-017-4318-z 28776081

[B51] Bonnet F. Scheen A. (2017). Understanding and Overcoming Metformin Gastrointestinal Intolerance. Diabetes Obes. Metab. 19 (4), 473–481. 10.1111/dom.12854 27987248

[B49] Bouchoucha M. Uzzan B. Cohen R. (2011). Metformin and Digestive Disorders. Diabetes Metab. 37 (2), 90–96. 10.1016/j.diabet.2010.11.002 21236717

[B3] Buse J. B. Rosenstock J. Sesti G. Schmidt W. E. Montanya E. Brett J. H. (2009). Liraglutide Once a Day Versus Exenatide Twice a Day for Type 2 Diabetes: A 26-Week Randomised, Parallel-Group, Multinational, Open-Label Trial (Lead-6). Lancet 374 (9683), 39–47. 10.1016/s0140-6736(09)60659-0 19515413

[B41] Castillo-Quan J. I. Blackwell T. K. (2016). Metformin: Restraining Nucleocytoplasmic Shuttling to Fight Cancer and Aging. Cell 167 (7), 1670–1671. 10.1016/j.cell.2016.11.058 27984715

[B32] Charbonnel B. Karasik A. Liu J. Wu M. Meininger G. (2006). Efficacy and Safety of the Dipeptidyl Peptidase-4 Inhibitor Sitagliptin Added to Ongoing Metformin Therapy in Patients With Type 2 Diabetes Inadequately Controlled With Metformin Alone. Diabetes Care 29 (12), 2638–2643. 10.2337/dc06-0706 17130197

[B18] Chen C. Morris S. J. I. C. S. (2003). Visualizing Evolving Networks: Minimum Spanning Trees Versus Pathfinder Networks. Seattle, WA: IEEE.

[B14] Chen C. (2006). Citespace II: Detecting and Visualizing Emerging Trends and Transient Patterns In Scientific Literature. J. Am. Soc. Inf. Sci. 57 (3), 359–377. 10.1002/asi.20317

[B13] Chen C. (2004). Searching for Intellectual Turning Points: Progressive Knowledge Domain Visualization. Proc. Natl. Acad. Sci. 101 (Suppl 1), 5303–5310. 10.1073/pnas.0307513100 14724295 PMC387312

[B40] Coll A. P. Chen M. Taskar P. Rimmington D. Patel S. Tadross J. A. (2020). Gdf15 Mediates the Effects of Metformin on Body Weight and Energy Balance. Nature 578 (7795), 444–448. 10.1038/s41586-019-1911-y 31875646 PMC7234839

[B37] Decensi A. Puntoni M. Goodwin P. Cazzaniga M. Gennari A. Bonanni B. (2010). Metformin and Cancer Risk in Diabetic Patients: A Systematic Review and Meta-Analysis. Cancer Prev. Res. 3 (11), 1451–1461. 10.1158/1940-6207.capr-10-0157 20947488

[B31] DeFronzo R. A. Ratner R. E. Han J. Kim D. D. Fineman M. S. Baron A. D. (2005). Effects of Exenatide (Exendin-4) on Glycemic Control and Weight Over 30 Weeks in Metformin-Treated Patients With Type 2 Diabetes. Diabetes Care 28 (5), 1092–1100. 10.2337/diacare.28.5.1092 15855572

[B48] Dowling R. J. O. Zakikhani M. Fantus I. G. Pollak M. Sonenberg N. (2007). Metformin Inhibits Mammalian Target of Rapamycin-Dependent Translation Initiation in Breast Cancer Cells. Cancer Res. 67 (22), 10804–10812. 10.1158/0008-5472.can-07-2310 18006825

[B29] Drucker D. J. Nauck M. A. (2006). The Incretin System: Glucagon-Like Peptide-1 Receptor Agonists and Dipeptidyl Peptidase-4 Inhibitors in Type 2 Diabetes. Lancet 368 (9548), 1696–1705. 10.1016/s0140-6736(06)69705-5 17098089

[B8] Foretz M. Guigas B. Bertrand L. Pollak M. Viollet B. (2014). Metformin: From Mechanisms of Action to Therapies. Cell Metab. 20 (6), 953–966. 10.1016/j.cmet.2014.09.018 25456737

[B7] Foretz M. Guigas B. Viollet B. (2019). Understanding the Glucoregulatory Mechanisms of Metformin in Type 2 Diabetes Mellitus. Nat. Rev. Endocrinol. 15 (10), 569–589. 10.1038/s41574-019-0242-2 31439934

[B39] Forslund K. Hildebrand F. Hildebrand F. Nielsen T. Falony G. Le Chatelier E. (2015). Disentangling Type 2 Diabetes and Metformin Treatment Signatures in the Human Gut Microbiota. Nature 528 (7581), 262–266. 10.1038/nature15766 26633628 PMC4681099

[B33] Fryer L. G. D. Parbu-Patel A. Carling D. (2002). The Anti-Diabetic Drugs Rosiglitazone and Metformin Stimulate Amp-Activated Protein Kinase Through Distinct Signaling Pathways. J. Biol. Chem. 277 (28), 25226–25232. 10.1074/jbc.m202489200 11994296

[B30] Garber A. Henry R. Ratner R. Garcia-Hernandez P. A. Rodriguez-Pattzi H. Olvera-Alvarez I. (2009). Liraglutide Versus Glimepiride Monotherapy For Type 2 Diabetes (Lead-3 Mono): A Randomised, 52-Week, Phase III, Double-Blind, Parallel-Treatment Trial. Lancet 373 (9662), 473–481. 10.1016/s0140-6736(08)61246-5 18819705

[B17] Garfield E. (1999). Journal. impact Factor a Brief Review. CMAJ 161 (8), 979–980. 10551195 PMC1230709

[B12] Garfield E. J. S. (1987). Citation Data is Subtle Stuff. Scientist-Mag. Life Sci. 1, 9

[B22] International Diabetes Federation Guideline Development Group (2014). Global guideline for type 2 diabetes. Diabetes Res. Clin. Pract. 104 (1), 1–52. 10.1016/j.diabres.2012.10.001 24508150

[B1] Graham G. G. Punt J. Arora M. Day R. O. Doogue M. P. Duong J. K. (2011). Clinical Pharmacokinetics of Metformin. Clin. Pharmacokinet. 50 (2), 81–98. 10.2165/11534750-000000000-00000 21241070

[B16] Hirsch J. E. (2005). An Index to Quantify an Individual’s Scientific Research Output. Proc. Natl. Acad. Sci. 102 (46), 16569–16572. 10.1073/pnas.0507655102 16275915 PMC1283832

[B23] Holman R. R. Paul S. K. Bethel M. A. Matthews D. R. Neil H. A. W. (2008). 10-Year Follow-Up of Intensive Glucose Control in Type 2 Diabetes. N. Engl. J. Med. 359 (15), 1577–1589. 10.1056/nejmoa0806470 18784090

[B46] Kahn S. E. Haffner S. M. Heise M. A. Herman W. H. Holman R. R. Jones N. P. (2006). Glycemic Durability of Rosiglitazone, Metformin, or Glyburide Monotherapy. N. Engl. J. Med. 355 (23), 2427–2443. 10.1056/nejmoa066224 17145742

[B27] Kalender A. Selvaraj A. Kim S. Y. Gulati P. Brûlé S. Viollet B. (2010). Metformin, Independent of Ampk, Inhibits Mtorc1 in a Rag Gtpase-Dependent Manner. Cell Metab. 11 (5), 390–401. 10.1016/j.cmet.2010.03.014 20444419 PMC3081779

[B44] Knowler W. C. Barrett-Connor E. Fowler S. E. Hamman R. F. Lachin J. M. Walker E. A. (2002). Reduction in the Incidence of Type 2 Diabetes With Lifestyle Intervention or Metformin. N. Engl. J. Med. 346 (6), 393–403. 10.1056/NEJMoa012512 11832527 PMC1370926

[B5] Kulkarni A. S. Gubbi S. Barzilai N. (2020). Benefits of Metformin in Attenuating the Hallmarks of Aging. Cell Metab. 32 (1), 15–30. 10.1016/j.cmet.2020.04.001 32333835 PMC7347426

[B15] Lin H. Zhu Y. Ahmad N. Han Q. (2019). A Scientometric Analysis and Visualization of Global Research on Brownfields. Environ. Sci. Pollut. Res. 26 (17), 17666–17684. 10.1007/s11356-019-05149-3 31028623

[B26] Madiraju A. K. Erion D. M. Rahimi Y. Zhang X. M. Braddock D. T. Albright R. A. (2014). Metformin Suppresses Gluconeogenesis by Inhibiting Mitochondrial Glycerophosphate Dehydrogenase. Nature 510 (7506), 542–546. 10.1038/nature13270 24847880 PMC4074244

[B9] McCreight L. J. Bailey C. J. Pearson E. R. (2016). Metformin and the Gastrointestinal Tract. Diabetologia 59 (3), 426–435. 10.1007/s00125-015-3844-9 26780750 PMC4742508

[B36] Moghetti P. Castello R. Negri C. Tosi F. Perrone F. Caputo M. (2000). Metformin Effects on Clinical Features, Endocrine and Metabolic Profiles, and Insulin Sensitivity in Polycystic Ovary Syndrome: A Randomized, Double-Blind, Placebo-Controlled 6-Month Trial, Followed by Open, Long-Term Clinical Evaluation1. J. Clin. Endocrinol. Metab. 85 (1), 139–146. 10.1210/jcem.85.1.6293 10634377

[B25] Musi N. Hirshman M. F. Nygren J. Svanfeldt M. Bavenholm P. Rooyackers O. (2002). Metformin Increases Amp-Activated Protein Kinase Activity in Skeletal Muscle of Subjects With Type 2 Diabetes. Diabetes 51 (7), 2074–2081. 10.2337/diabetes.51.7.2074 12086935

[B20] Nattrass M. Alberti K. G. M. M. (1978). Biguanides. Diabetologia 14 (2), 71–74. 10.1007/bf01263443 631459

[B34] Nissen S. E. Wolski K. (2007). Effect of Rosiglitazone on the Risk of Myocardial Infarction and Death from Cardiovascular Causes. N. Engl. J. Med. 356 (24), 2457–2471. 10.1056/nejmoa072761 17517853

[B10] Pritchard A. (1969). Stat. Bibliography or Bibliometrics? J. Doc 25 (4), 348–349.

[B38] Pryor R. Norvaisas P. Marinos G. Best L. Thingholm L. B. Quintaneiro L. M. (2019). Host-Microbe-Drug-Nutrient Screen Identifies Bacterial Effectors of Metformin Therapy. Cell 178 (6), 1299–1312.e29. 10.1016/j.cell.2019.08.003 31474368 PMC6736778

[B47] Rena G. Hardie D. G. Pearson E. R. (2017). The Mechanisms of Action of Metformin. Diabetologia 60 (9), 1577–1585. 10.1007/s00125-017-4342-z 28776086 PMC5552828

[B19] Sanchez-Rangel E. Inzucchi S. E. (2017). Metformin: Clinical Use in Type 2 Diabetes. Diabetologia 60 (9), 1586–1593. 10.1007/s00125-017-4336-x 28770321

[B6] Scheen A. J. (2020). Metformin and Covid-19: From Cellular Mechanisms to Reduced Mortality. Diabetes Metab. 46 (6), 423–426. 10.1016/j.diabet.2020.07.006 32750451 PMC7395819

[B28] Shaw R. J. Lamia K. A. Vasquez D. Koo S. H. Bardeesy N. Depinho R. A. (2005). The Kinase Lkb1 Mediates Glucose Homeostasis in Liver and Therapeutic Effects of Metformin. Science 310 (5754), 1642–1646. 10.1126/science.1120781 16308421 PMC3074427

[B50] Shu Y. Sheardown S. A. Brown C. Owen R. P. Zhang S. Castro R. A. (2007). Effect of Genetic Variation in the Organic Cation Transporter 1 (Oct1) on Metformin Action. J. Clin. Invest. 117 (5), 1422–1431. 10.1172/jci30558 17476361 PMC1857259

[B42] Sivalingam V. McVey R. Gilmour K. Ali S. Roberts C. Renehan A. (2015). A Presurgical Window-of-Opportunity Study of Metformin in Obesity-Driven Endometrial Cancer. Lancet 385 (Suppl. 1), S90. 10.1016/s0140-6736(15)60405-6 26312913

[B11] Thompson D. F. Walker C. K. (2015). A Descriptive and Historical Review of Bibliometrics with Applications to Medical Sciences. Pharmacotherapy 35 (6), 551–559. 10.1002/phar.1586 25940769

[B80] World Health Organization (2019). WHO Model List of Essential Medicines www.who.int/groups/expert-committee-on-selection-and-use-of-essential-medicines/essential-medicines-lists (Accessed April 20, 2021).

[B45] Zakikhani M. Dowling R. Fantus I. G. Sonenberg N. Pollak M. (2006). Metformin is an Amp Kinase-Dependent Growth Inhibitor for Breast Cancer Cells. Cancer Res. 66 (21), 10269–10273. 10.1158/0008-5472.can-06-1500 17062558

[B2] Zilov A. V. Abdelaziz S. I. AlShammary A. Al Zahrani A. Amir A. Assaad Khalil S. H. (2019). Mechanisms of Action of Metformin With Special Reference to Cardiovascular Protection. Diabetes/Metabolism Res. Rev. 35 (7), e3173. 10.1002/dmrr.3173 PMC6851752 31021474

[B35] Zinman B. Wanner C. Lachin J. M. Fitchett D. Bluhmki E. Hantel S. (2015). Empagliflozin, Cardiovascular Outcomes, and Mortality in Type 2 Diabetes. N. Engl. J. Med. 373 (22), 2117–2128. 10.1056/nejmoa1504720 26378978

